# Mapping a Circular RNA–microRNA–mRNA-Signaling Regulatory Axis that Modulates Stemness Properties of Cancer Stem Cell Populations in Colorectal Cancer Spheroid Cells

**DOI:** 10.3390/ijms21217864

**Published:** 2020-10-23

**Authors:** Vimalan Rengganaten, Chiu-Jung Huang, Ping-Hsing Tsai, Mong-Lien Wang, Yi-Ping Yang, Yuan-Tzu Lan, Wen-Liang Fang, Shelly Soo, Hooi Tin Ong, Soon Keng Cheong, Kong Bung Choo, Shih-Hwa Chiou

**Affiliations:** 1 Centre for Stem Cell Research, Universiti Tunku Abdul Rahman, Kajang 43000, Malaysia; vimalanrengganaten@gmail.com (V.R.); onght@utar.edu.my (H.T.O.); cheongsk@utar.edu.my (S.K.C.); 2 Postgraduate Programme, Department of Preclinical Sciences, Faculty of Medicine and Health Sciences, Universiti Tunku Abdul Rahman, Kajang 43000, Malaysia; 3 Institute of Pharmacology, National Yang-Ming University, Taipei 11221, Taiwan; figatsai@gmail.com (P.-H.T.); molly0103@gmail.com (Y.-P.Y.); 4 Department of Animal Science & Graduate Institute of Biotechnology, Chinese Culture University, Taipei 11221, Taiwan; hqr2@faculty.pccu.edu.tw; 5 Department of Medical Research, Taipei Veterans General Hospital, Taipei 11221, Taiwan; monglien@gmail.com; 6 Institute of Food Safety and Health Risk Assessment, National Yang-Ming University, Taipei 11221, Taiwan; 7 School of Pharmaceutical Sciences, National Yang-Ming University, Taipei 11221, Taiwan; 8 Faculty of Medicine, National Yang-Ming University, Taipei 11221, Taiwan; ytlan@vghtpe.gov.tw (Y.-T.L.); wlfang@vghtpe.gov.tw (W.-L.F.); 9 Department of Colorectal Surgery, Taipei Veterans General Hospital, Taipei 11221, Taiwan; 10 Department of Surgery, Taipei Veterans General Hospital, Taipei 11221, Taiwan; 11 Gastric Cancer Medical Center, Taipei Veterans General Hospital, Taipei 11221, Taiwan; 12 Bariatric and Metabolic Surgery Center, Taipei Veterans General Hospital, Taipei 11221, Taiwan; 13 Centre for Research on Non-Communicable Diseases, Universiti Tunku Abdul Rahman, 43000 Kajang, Malaysia; shellysoo@ymail.com; 14 Department of Clinical Sciences, Faculty of Medicine and Health Sciences, Universiti Tunku Abdul Rahman, 43000 Kajang, Malaysia; 15 Department of Preclinical Sciences, Faculty of Medicine and Health Sciences, Universiti Tunku Abdul Rahman, 43000 Kajang, Malaysia; 16 Dean’s Office, Faculty of Medicine and Health Sciences, Universiti Tunku Abdul Rahman, 43000 Kajang, Malaysia

**Keywords:** colorectal cancer, spheroid culture, colorectal cancer stem cells, circular RNA, microRNA, stemness

## Abstract

Spheroidal cancer cell cultures have been used to enrich cancer stem cells (CSC), which are thought to contribute to important clinical features of tumors. This study aimed to map the regulatory networks driven by circular RNAs (circRNAs) in CSC-enriched colorectal cancer (CRC) spheroid cells. The spheroid cells established from two CRC cell lines acquired stemness properties in pluripotency gene expression and multi-lineage differentiation capacity. Genome-wide sequencing identified 1503 and 636 circRNAs specific to the CRC parental and spheroid cells, respectively. In the CRC spheroids, algorithmic analyses unveiled a core network of mRNAs involved in modulating stemness-associated signaling pathways, driven by a circRNA–microRNA (miRNA)–mRNA axis. The two major circRNAs, hsa_circ_0066631 and hsa_circ_0082096, in this network were significantly up-regulated in expression levels in the spheroid cells. The two circRNAs were predicted to target and were experimentally shown to down-regulate miR-140-3p, miR-224, miR-382, miR-548c-3p and miR-579, confirming circRNA sponging of the targeted miRNAs. Furthermore, the affected miRNAs were demonstrated to inhibit degradation of six mRNA targets, viz. ACVR1C/ALK7, FZD3, IL6ST/GP130, SKIL/SNON, SMAD2 and WNT5, in the CRC spheroid cells. These mRNAs encode proteins that are reported to variously regulate the GP130/Stat, Activin/Nodal, TGF-β/SMAD or Wnt/β-catenin signaling pathways in controlling various aspects of CSC stemness. Using the CRC spheroid cell model, the novel circRNA–miRNA–mRNA axis mapped in this work forms the foundation for the elucidation of the molecular mechanisms of the complex cellular and biochemical processes that determine CSC stemness properties of cancer cells, and possibly for designing therapeutic strategies for CRC treatment by targeting CSC.

## 1. Introduction

Cancer treatment faces two major challenges, viz. recurrence and metastasis, both attributed to the presence of cancer stem cells (CSCs) in the tumor [1]. CSCs are a small cell subpopulation in a tumor that have been shown to act as the driver of tumor progression and metastasis [2]. The cell of origin of CSCs remains controversial, with possibilities of CSCs arising from either stem cells or non-stem cells [3,4]. In a postulated “bottom-up” model of the genesis of colorectal cancer (CRC), CRC stem cells (CrCSCs) are thought to arise from the intestinal stem cells residing at the bottom of the colonic crypts due to the accumulation of mutations; CrCSCs harboring the mutations subsequently repopulate the crypts, leading to tumor formation [5,6,7].

The unique properties of CSCs could arise from mutation-conferred oncogenic properties, while generally maintaining the intrinsic characteristics of stem cells. Hence, the unique features of CSCs include self-renewal, multi-lineage differentiation abilities, dysregulated proliferation control, enhanced tumorigenicity and chemo- and radio-therapeutic resistance [8,9,10]. To target and eradicate CSCs from the tumor mass, as has been attempted in differentiation therapy [4,11], it is important to first understand the regulatory mechanisms of stemness properties of CSCs population. To circumvent the sparsity of CSC population, spheroid culture has been used to enrich CSCs [12,13,14]. Spheroid culture is an anchorage-independent three-dimensional culture that mimics in vivo microenvironment, reduces cell–matrix interactions to achieve positive selection of cells that are in higher levels of hierarchy via anoikis-induced apoptosis [15]. Apart from demonstrating higher tumorigenicity in in vivo models [16,17], spheroid cells exhibit CSC-like features, including enhanced chemoresistance, metastatic and differentiation ability [18,19,20,21]. 

Genome-wide next-generation sequencing and bioinformatics analysis of RNA sequences have led to the discovery of a novel class of epigenetic regulators, called circular RNA (circRNA). CircRNAs are long non-coding RNAs that have been reported to regulate major cancer-related biological processes, including tumorigenicity and chemoresistance [22,23,24,25]. Derived from back-splicing of primary transcripts of mainly protein-coding genes, circRNAs may consist of exons or introns, or a mixture of both exons and introns [26]. The covalently closed loop of the 3′ and 5′ ends of circRNAs confers higher stability and longer half-lives to circRNAs; since circRNAs are more likely to be detected in bodily fluid, circRNAs are being developed as cancer or disease biomarkers [27,28]. Functionally, circRNAs regulate the expression of the parental host mRNA via competition [29,30]. However, a more important function of circRNAs is to act as sponges of microRNAs (miRNAs), another class of well-characterized negative regulators of gene expression [31]. Furthermore, circRNAs also act as protein sponges to more directly modulate protein functions [32]. As miRNA sponges, circRNAs reduce the availability of miRNAs to interact with downstream target mRNAs and, thereby, circRNA indirectly regulates mRNA expression [33,34]. By sponging with proteins, particularly RNA-binding proteins, circRNA sequesters and modulates protein–protein interaction [35,36]. Hence, dysregulated expression of circRNAs could lead to the development of various diseases, including neoplasm [37].

To elucidate the regulatory role of circRNA, it is important to first map the circRNA–miRNA–mRNA axis, or regulatory network, of the circRNAs being investigated. In this work, we aimed to elucidate the regulatory and molecular mechanisms of stemness in colorectal cancer stem cells. To achieve this goal, we first generated CRC cells that exhibit enhanced stemness features via spheroid culture of CRC cell lines. The spheroid cells were then subjected to genome-wide high-throughput RNA sequencing targeting circRNAs. The differentially expressed circRNAs in the spheroid cells were subsequently used in the construction of circRNA–miRNA–mRNA regulatory networks. Here, we describe a novel circRNA–miRNA–mRNA network of multiple signaling pathways that regulates pathways mediating stemness in CRC spheroids.

## 2. Results

### 2.1. Generation of CRC Spheroid Cells That Showed Enhanced Stemness-Related Properties

**Figure 1:**
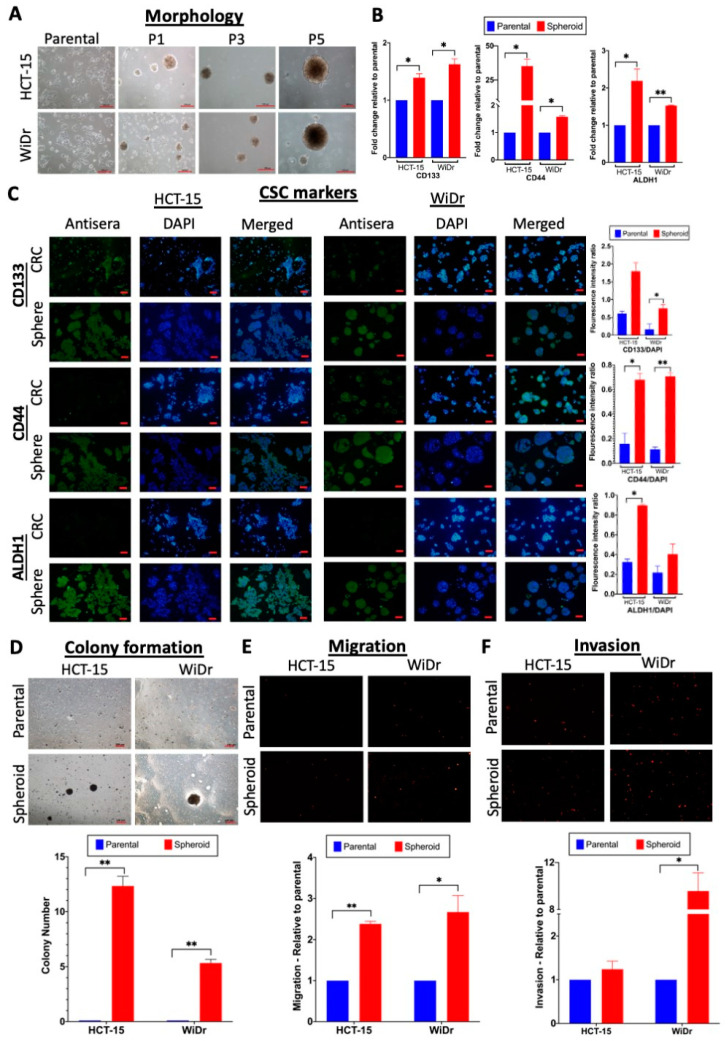
Generation of cancer stem cell (CSC)-enriched colorectal cancer (CRC) spheroid cells that showed enhanced stemness-related properties. (**A**) Morphology of CRC parental and spheroid cells at different passages (P) in the spheroid culture (bars: 100 μm). (**B**,**C**) Up-regulated expression of CSC markers CD133, CD44 and aldehyde dehydrogenase 1 (ALDH1). (**B**) qRT-PCR analysis of CSC markers on parental and spheroid cells. Glyceraldehyde 3-phosphate dehydrogenase (GAPDH) was used to normalize the expression levels. (**C**) Immunofluorescence analysis of CSC markers was performed using specific antisera (green). Staining with DAPI (blue) and merged images are also shown. Quantitative analysis is shown as bar graph, relative to the DAPI expression levels. (**D**) Enhanced colony formation ability of the spheroid cells. Images of cells after 10 days of culture in semi-solid medium are shown (bar: 100 μm). Quantitative analysis of number of colonies >100 μm in size is shown in the bottom panel based on three independent experiments. (**E**,**F**) Enhanced migration and invasion properties of the spheroid cells in transwell assays. Fluorescence images of migrated (**E**) and invaded (**F**) cells in the transwell chambers. In the qualitative analysis relative to values of the parental cells, arbitrarily set at 1.0 (bottom panels), * *p* < 0.05 and ** *p* < 0.01 were relative to the values of the parental cells.

To generate colorectal cancer (CRC) spheroids, two CRC cell lines, HCT-15 and WiDr, both derived from colorectal adenocarcinoma, were cultured in a serum-free medium supplemented with growth factors on polyhydroxyethylmethacrylate (polyHEMA)-coated culture flasks. The CRC cells grew as a monolayer in culture and showed epithelial cell-like morphology. Under spheroid culture conditions, however, the cells formed clumps, which expanded in size over a duration of up to 14 days of culture (Figure 1A). When the spheroids were passaged every 10–14 days, new generations of spheroids were formed under the same suspension culture conditions. The CRC spheroids formed generally became more spherical in shape and larger in size on further passaging of the cells (Figure 1A), indicating passage-dependent enhancement of self-renewal ability, an important feature of stem cells [38]. To evaluate the enrichment of cancer stem cells (CSC) in the spheroids, the expression of three known CSC markers, CD133, CD44 and aldehyde dehydrogenase 1 (ALDH1), was analyzed using qRT-PCR and immunofluorescence (Figure 1B,C). All of the CSC markers were significantly up-regulated in the spheroid cells relative to the parental cells, indicative of enriched CSC populations in the spheroids.

To further investigate other stem cell-like properties, the self-renewal ability of the spheroids was further tested in colony-forming assays by culturing the spheroid-derived single-cell suspension under anchorage-independent conditions in semi-solid media. The number of colonies larger than 100 μm was counted after 10 days. The CRC cells survived and proliferated in the semi-solid medium; on the other hand, the CRC spheroids significantly expanded in volume, as reflected in the increase in the number of colonies >100 μm in size (Figure 1D), indicating higher self-renewal abilities. The CRC spheroid cells also showed significantly higher migration and invasion abilities than the parental CRC cells in transwell assays (Figure 1E,F), further demonstrating enrichment of a CSC-like phenotype in the spheroids.

### 2.2. Differentiation Abilities and Chemoresistance of the CRC Spheroid CSC Cells

**Figure 2:**
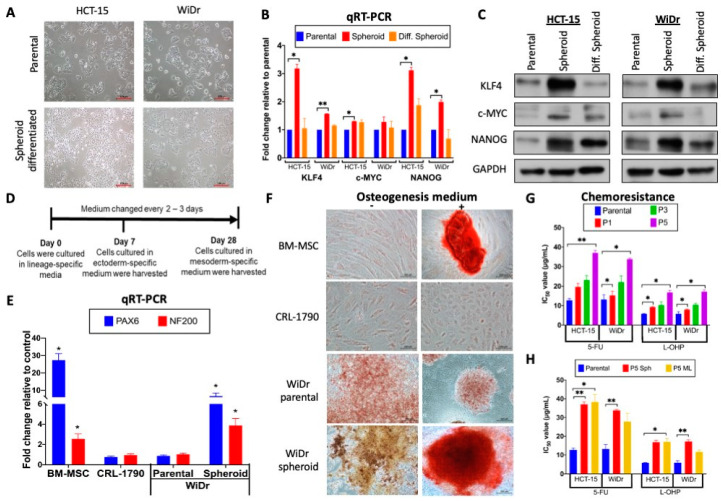
Differentiation abilities and chemoresistance of the CRC spheroid cells. (**A**) Serum-induced differentiation reverted the morphology of the spheroid cells to that of the parental CRC cells. Induced differentiation was achieved by culturing the CRC spheroid cells in a serum-containing medium (see Materials and Methods), and the morphologies of the cells are shown (bars: 100 μm). (**B**) Up-regulated expression of pluripotency genes in the CRC spheroids and in serum-induced differentiated cells analyzed in (**B**) qRT-PCR and (**C**) western blots. (**D**–**F**) Lineage-directed differentiation of the CRC spheroid cells. (**D**) Timeline and culture conditions of the lineage-directed differentiation experiments. The ectoderm-directed differentiation was achieved in 7 days of culture before the cells were harvested for qRT-PCR analysis (**E**); mesoderm-directed differentiation was analyzed by staining after 28 days (**F**). (**E**) Ectodermic differentiation of the WiDr spheroid cells. Relative expression levels of the ectoderm-specific markers, PAX6 and NF-200, in WiDr spheroids and WiDr spheroid-differentiated cells relative to non-cancerous colonic CRL-1790 cells, used as the controls, are shown. The bone marrow-derived mesenchymal stem cells (BM-MSC) were included as a positive control. The expression levels were normalized to those of CRL-1790, set as 1.0. (**F**) Mesodermic differentiation of the CRC spheroid cells. CRC spheroid cells were differentiated into the mesoderm-derived osteocytes visualized by Alizarin Red staining on day 28. (bars: 100 μm). The control was cells cultured in normal media. (**G**,**H**) Progressive enhancement of chemoresistance in CRC spheroids on extended culture. After treatment with 5-fluorouracil (5-FU) or oxaliplatin (L-OHP) at a concentration range of 0 to 100 μg/mL and at different passages (P1, P3 and P5) of the spheroids, IC_50_ values of the cells were obtained by MTT assays (**G**). Drug treatment was similarly performed on P5 monolayer (P5-ML) cells derived from the P5 spheroids (P5 Sph) (**H**). In all subfigures, * *p* < 0.05 and ** *p* < 0.01 were values relative to the similarly treated parental CRC cells.

A key feature of cellular stemness is the ability to differentiate, and serum has been reported to act as an inducing agent for differentiation of stem cells [39]. To test the differentiation ability of the CRC spheroid cells, the spheroids were subjected to serum-induced differentiation by culturing passage 5 (P5) spheroids in a serum-containing medium. The differentiated spheroid cells showed a morphology highly resembles that of the parental cells (Figure 2A), indicative of having undergone differentiation. Furthermore, the differentiated cells were able to survive and stably proliferate to up to 10 passages as monolayers (data not shown). CSC stemness has been characterized by the expression of pluripotency-associated stemness transcriptional factors, typically KLF4, c-MYC and NANOG [40,41], while Western blot and qRT-PCR analysis indicated up-regulated expression of these stemness factors in the spheroid cells relative to the parental CRC cells; the up-regulation was reversed in the serum-induced differentiated cells (Figure 2B,C), consistent with spheroid culture-dependent stemness enrichment. 

Noting the up-regulated expression of the pluripotency-associated transcriptional factors, the differentiation potentials of the spheroids were next tested in lineage-directed differentiation media. Since the colon is an organ derived from the endoderm lineage, the WiDr spheroids were tested for their ability to differentiate into the ectodermic and mesodermic lineages. Bone marrow-derived mesenchymal stem cells (BM-MSC) and human non-malignant colonic CRL-1790 cells were used as controls using the timeline and culture conditions shown in Figure 2D. In the ectoderm lineage differentiation test, PAX6 and NF-200, markers of the ectoderm-derived neuron, were shown to be up-regulated in the WiDr spheroids as in the BM-MSC control, indicating ectoderm lineage differentiation (Figure 2E). In mesoderm lineage differentiation assays, Alizarin Red S staining of the WiDr spheroids and BM-MSC indicated differentiation into the mesoderm-derived osteocytes, but not in CRL-1790 nor the parental WiDr cells (Figure 2F). Taken together, the data showed that the WiDr spheroid cells had acquired a multi-lineage differentiation capacity. It is important to note here that, under the same differentiation conditions, HCT-15 cells detached and did not survive the prolonged culture (data not shown), and were not subjected to lineage-directed differentiation assays.

A hallmark of CSC is enhanced chemoresistance [42]. Hence, the chemoresistance properties of the HCT-15- and WiDr-derived spheroid cells to the two commonly used CRC chemotherapeutic drugs, 5-fluorouracil (5-FU) and oxaliplatin (L-OHP), were evaluated in MTT assays at 0–100 μg/mL drug concentrations. Spheroids of both cell lines showed higher IC_50_ values, in a passage-dependent manner from passages P1 to P5, after drug treatment compared to the parental cells, confirming progressive enhancement of chemoresistance (Figure 2G). To investigate if the three dimensional (3D) structure of the spheroids abated drug entry [43], drug sensitivity assays were also performed by first dissociating P5 spheroids into a single-cell suspension. The cells were subsequently cultured as a monolayer for 24 h before drug treatment at the same drug concentration range under the same culture conditions as for the CRC cells (Figure 2H). The results also showed enhanced chemoresistance in the spheroid-derived monolayer cells, indicating that the CRC cells in the spheroids had, indeed, acquired enhanced chemoresistance, ruling out structural hindrances on drug entry. Collectively, the CRC spheroid cells showed multiple hallmark features of cancer stem cells and exhibited higher stemness properties.

### 2.3. Genome-Wide Expression Profiling of circRNAs in the CSC-Like Cells in the CRC Spheroids

**Figure 3:**
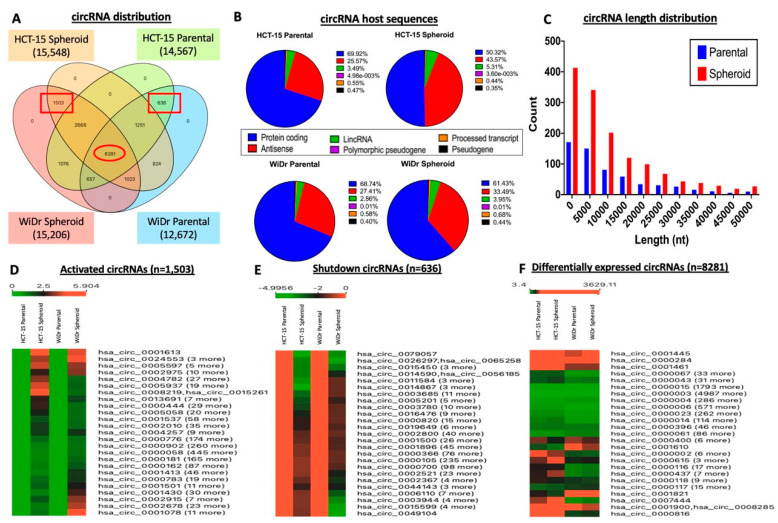
Genome-wide expression profiling of circRNAs in the CRC spheroid CSC cells. (**A**) Overlapping circRNAs identified in the CRC and spheroid CSC cells. The numbers in red boxes indicate circRNAs that were either activated or up-regulated (left red box), or shutdown or down-regulated (right red box), and the red oval box indicates circRNAs that were differentially expressed in the spheroids relative to the parental CRC cells. (**B**,**C**) Distribution of the sources of the host transcripts (**B**) and length distribution (**C**) of the differentially expressed circular RNAs. The results shown are combined data of the two cell lines. nt, nucleotide. (**D**–**F**) Hierarchical clustering analysis of activated (**D**) and shutdown (**E**) circRNAs, in terms of activation and shutdown from and to zero expression level, respectively (see also square boxes in Figure 3A), and differentially expressed circRNAs (F; see also the oval box in Figure 3A) in the spheroids relative to the CRC cells. Apart from a representative circRNA shown in its designation in an expression-level cluster, the total number (n) of circRNAs in each expression cluster is also shown in brackets.

To identify circRNAs that are altered in expression levels during the process of spheroid formation, NGS genome-wide circRNA sequencing of RNase R-treated RNA samples of HCT-15 and WiDr and the derived spheroid cells was performed. The results showed that ~15,000 circRNAs were collectively detected with different circRNA distribution patterns in the parental and spheroid cells (Figure 3A). Notably, 1503 circRNAs were found to be commonly expressed in the spheroid cells generated from the two cell lines, which are not expressed in the cancer parental cells (Figure 3A, left-hand red box); these circRNAs were activated on spheroidal-culture reprogramming of the CRC cells. Likewise, 636 circRNAs were found only in CRC but not in the spheroid cells (Figure 3A, right-hand red box), probably representing circRNAs that were inactivated or shutdown in the spheroids. 

Analysis of the host transcripts of the differentially expressed circRNAs revealed that most of the circRNAs were derived from protein-coding RNA: 50.32% and 61.43% in HCT-15 and WiDr spheroids, respectively (Figure 3B). Notably, on spheroid formation, the number of circRNAs derived from protein-coding transcripts decreased: HCT-15 decreased by 19.6% and WiDr by 7.1%, to antisense and long intervening non-coding RNAs (lincRNA). Derivation from antisense RNAs increased from 25.57% to 43.57% and 27.41% to 33.49% in HCT-15 and WiDr spheroids, respectively, and that from lincRNAs increased from 3.49% to 5.31% and 2.88% to 3.96% for the two cell lines. As circRNAs may act as a competitive regulator of its linear RNA host transcript [44], decreased utilization of protein-coding transcripts in the derivation of circRNAs could mean a decreased regulatory role for circRNAs in modulating the expression of protein-coding genes, and that circRNAs may play a more prominent role in regulating the biological functions of antisense and lincRNAs. Analysis of length distribution of the circRNAs showed a ~two-fold increase in the abundance of circRNAs in the spheroids (Figure 3C), an observation consistent with a higher combined number of circRNAs in spheroids (1503) than in the CRC cells (636) (Figure 3A). Furthermore, circRNAs from 5000 to 20,000 nucleotides in length were most abundant (Figure 3C); however, the biological implications of the observed circRNA population and length re-distribution in the spheroidal CSC cells remain to be investigated. 

Hierarchical clustering analysis based on circRNA expression levels showed that there were 1503 that were commonly activated or up-regulated, and 636 circRNAs shut down or down-regulated in the spheroids (Figure 3D,E; see also square boxes in Figure 3A) in terms of up-regulation from zero expression level (activation) and down-regulation to zero level (shutdown), respectively, in the spheroids compared with the parental cells. Further analysis also identified 8281 circRNAs (Figure 3F; see oval box in Figure 3A) that were differentially expressed, as opposed to activation or shutdown, in both the HCT-15 and WiDr spheroid cells.

### 2.4. The Top Eight Differentially Expressed circRNAs Form A Core circRNA–miRNA–mRNA Network to Regulate CSC Stemness Properties

**Figure 4:**
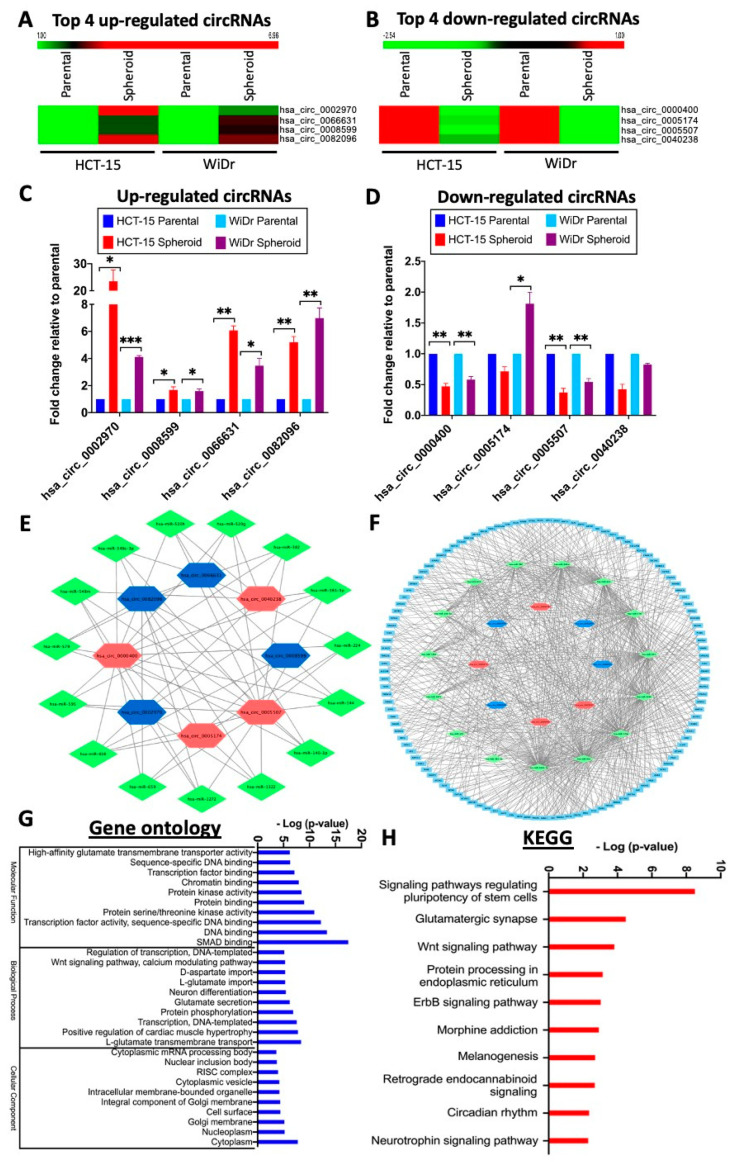
The top eight differentially expressed circular RNAs (circRNAs) form a core circRNA–microRNA (miRNA)–mRNA network to regulate stemness. (**A**,**B**) Hierarchical clustering analysis of top 4 up- (**A**) and down-regulated (**B**) circRNAs in the spheroid cells relative to the CRC cells. (**C**,**D**) Validation, by qRT-PCR, of the NGS expression data of the up- (**C**) or down-regulated (**D**) circRNAs in the spheroids; * *p* < 0.05, ** *p* < 0.01 and *** *p* < 0.001 were values relative to the parental cells. (**E**–**H**) A core circRNA–miRNA–mRNA network of the top eight differentially regulated circRNAs that regulate stemness of spheroid CSC cells. (**E**) The top four up- and down-regulated circRNAs and the predicted interacting miRNAs, selected by the criterion of four or more miRNA interactions with the circRNAs. Blue and red hexagons indicate up- or down-regulated circRNAs, respectively; green diamonds represent the interacting miRNAs (see also Supplementary Table S2 for further details). (**F**) Predicted mRNAs that interact with five or more of the predicted miRNAs in the circRNA–miRNA interaction network (see Suppl. Table S2). The mRNAs are shown in light blue boxes. (**G**,**H**) Top ten events identified in gene ontology (GO) (**G**) and Kyoto Encyclopedia of Genes and Genomes (KEGG) analyses (**H**) of the predicted interacting mRNAs identified in Figure 4F.

For further analysis, the top four up- and down-regulated circRNAs in both the HCT-15 and WiDr spheroid cells (Figure 4A,B; Table 1) were identified from the differentially expressed circRNA dataset of 8282 circRNAs since this dataset included expression values of both the parental and spheroidal cells of both the HCT-15 and WiDr cell lines (see also Figure 3A, red oval box, and Figure 3F). To authenticate the expression of these circRNAs, divergent backsplice junction primers specific to each circRNA (Supplementary Table S1; Supplementary Figure S1) were used in qRT-PCR analysis. The analysis confirmed that all of the top four up-regulated circRNAs were, indeed, significantly higher in their expression levels in the spheroids compared to the CRC cells (Figure 4C). In the down-regulated circRNA group, the circRNAs generally showed lower expression levels in the spheroids, except for circ_0005174, which was found to be up-regulated in the WiDr spheroids. It is noteworthy that, in the NGS profiling analysis, down-regulation of circ_0005174 was not statistically significant in both cell lines despite being one of the top four down-regulated circRNAs (Table 1).

A major biological function of circRNAs is to negatively regulate gene expression, via sponging the expression levels of microRNAs (miRNAs), which are themselves negative regulators, freeing the miRNA-targeted transcripts for translation [45]. To map the miRNA regulatory network driven by the top eight differentially expressed circRNAs, a search of the CircInteractome online database led to the identification of 279 interacting miRNAs (data not shown). When a more stringent criterion of four or more miRNA interactions with each circRNA was applied, fifteen miRNAs were identified (Figure 4E; see Supplementary Table S2 for interactions details). By applying the miRWalk algorithm, 4798 mRNAs were predicted to be targeted by the fifteen miRNAs (data not shown). The number of mRNAs was narrowed down to 121 by interrogating each transcript for interactions with five or more miRNAs (Figure 4F, Supplementary Table S3). Gene ontology (GO) analysis revealed that significant portions of the gene products of the mRNAs participate in binding activities of DNA, transcriptional factors and other regulatory proteins, including the SMAD proteins (Figure 4G). Biologically, the mRNAs are involved in DNA template-dependent transcription, protein phosphorylation and the Wnt signaling pathway, and the protein factors are primarily located in the cytoplasm where phosphorylation and signaling occur. In Kyoto Encyclopedia of Genes and Genomes (KEGG) analysis (Figure 4H), signaling pathways involved in regulating the pluripotency of stem cells were found to be significantly involved (Figure 4H), corroborating well with the cancer stem cell-like properties in the CRC spheroid cells described above (Figure 1 and Figure 2). Other implicated KEGG pathways included Wnt and ErbB signaling pathways, which have previously been associated with the regulation of the CrCSC population [46,47]. Hence, the mapping of circRNA–miRNA–mRNA profiling data predicted the involvement of signaling in stemness regulation.

**Figure 5:**
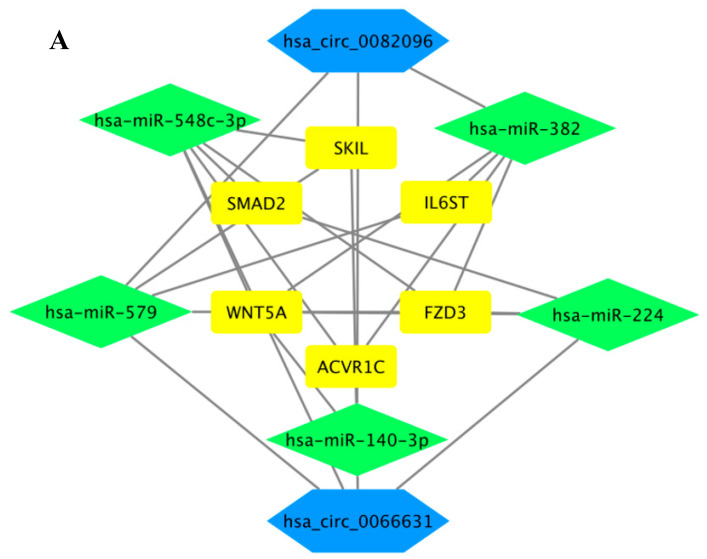

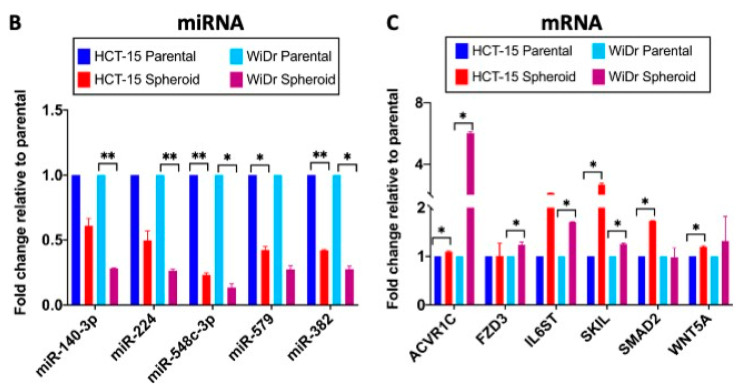
Circ_0082096 and circ_0066631 modulate stemness via a circRNA–miRNA–mRNA network. (**A**) CircRNAs (in hexagon), miRNA (in diamond) and mRNA (in box) that are predicted to regulate stemness. (**B**) Relative expression levels of the predicted miRNAs in the spheroid cells. Core miRNA analysis was performed by stem-loop qRT-PCR using small nucleolar RNA U6 as the normalization control. (**C**) Relative expression levels of predicted mRNA in the spheroid cells. Core mRNA analysis was by qRT-PCR using GAPDH as the control. * *p* < 0.05 and ** *p* < 0.01 were relative to the parental CRC cells (**B**,**C**).

Based on the KEGG analysis data (Figure 4H), further investigations were focused on pluripotency-signaling pathways in relation to possible regulation of the stemness of colorectal cancer stem cells, or CrCSC. Six core mRNAs, viz. ACVR1C/ALK7, FZD3, IL6ST/GP130, SKIL/SNON, SMAD2 and WNT5A, the gene products of which are involved in various pluripotency regulatory signaling pathways, were identified, mapping a core circRNA–miRNA–mRNA network of stemness regulation (Figure 5A). In the core network, the six mRNAs were collectively targeted by five core miRNAs, viz. miR-140-3p, miR-224, miR-382, miR-548c-3p and miR-579 (Table 2; see Supplementary Table S3 for full list), which, in turn, were under the control of two of the top up-regulated circRNAs, circ_0066631 and circ_0082096 identified above (Table 3). To obtain further supportive evidence on the predicted circRNA–miRNA interactions, circRNA–miRNA interaction sites were established based on minimal free energy values using the RNAhybrid prediction tool. The results showed that circ_0066631 and circ_0082096 were able to competitively bind with four and three, respectively, of the five miRNAs, whereas both the circRNAs were predicted to interact with miR-140-3p (Table 2). The interacting miRNAs shared a minimum of seven seed-complementary nucleotides on the circRNA sequence, with low predicted free energy between the complementary bases. 

To obtain experimental evidence to support the interactive map, the miRNA expression levels were assessed in the spheroids and cells derived from serum-induced differentiation of the spheroids using stem-loop qRT-PCR. All five miRNAs were significantly down-regulated in the spheroid cells (Figure 5B), which correlated well with up-regulated expression of circ_0066631 and circ_0082096 to suppress miRNA expression via miRNA sponging. Furthermore, upon differentiation, the miRNA expression levels reverted to the parental levels, or higher levels in some cases (Suppl. Figure S1A), further supporting the involvement of these miRNAs in the generation and maintenance of stemness in CrCSC. Likewise, the expression levels of the core mRNAs in the interactive map were analyzed using qRT-PCR. As anticipated from the suppressed miRNA expression patterns, the six core mRNAs were generally up-regulated in the spheroid cells (Figure 5C), supporting miRNA–mRNA interactions. However, upon serum-induced differentiation, only ACVRIC/ALK7 in HCT-15, SKIL/SNON (WiDr), SMAD2 (WiDr) and Wnt5A in both cell lines showed down-regulated expression whilst others were either up-regulated or showed no changes (Suppl. Figure S1B). Since miRNA is known to target multiple transcripts, and each transcript may be targeted by multiple miRNAs (Huang et al., 2014), true interactions between the core miRNAs and the transcripts in the proposed circRNA–miRNA–mRNA network remain to be further investigated. Nonetheless, bioinformatics analysis of the circRNA genome-wide profiling data had led to the mapping of a circRNA-driven circRNA–miRNA–mRNA regulatory network that plays an important role in regulating pluripotency-related biological process and, hence, stemness properties in the CRC spheroids generated.

## 3. Discussion

The acquisition of stemness properties in cancer cells could give rise to a subpopulation of cancer stem cells that exhibit multiple stem cell-like phenotypes [48]. To mimic tumor microenvironments and to enrich stem cell-like cells, a robust in vitro three-dimensional colorectal cancer spheroidal culture was developed and described in this work. In line with other works [49,50], the spheroidal culture showed passage-dependent phenotypic and molecular changes and displayed multiple features of stemness properties tested, including up-regulated expression of pluripotency factors, enhanced self-renewal, cell migration and invasion abilities, and multi-lineage differentiation (Figure 1 and Figure 2). Chemoresistance to 5-FU and oxaliplatin was enhanced in increasing passages of the spheroid cells, further supporting CSC enrichment in the spheroids (Figure 2F,G), as has also been reported for CRC and other cancers [51,52,53]. The ability of the cells to differentiate into ectodermic and mesodermic lineages supported the pluripotency of the CSC in the spheroids, as was also reported for CRC cancer stem cell-like population to differentiate into mucin-producing goblet cells, enterocyte-like and neuroendocrine-like cells [18]. The combined observation that the CRC spheroid cells readily differentiated into calcium-producing and central nervous system-type cells and that the cells showed enhanced migration and invasion properties could partly explain the propensity of CRC cells to metastasize to, and survive in the microenvironments of, bones and the central nervous system [54,55].

The discovery of circRNAs has added a new layer of complexity to the epigenetic-controlled regulation of gene expression primarily via the circRNA–miRNA–mRNA axis [56]. Possible involvements of circRNAs in the maintenance of CRC stemness in the CSC-enriched spheroids were investigated in this work via genome-wide circRNA profiling and algorithmic analysis. To focus on CSC stemness, two significantly up-regulated circRNAs, viz. circ_0066631 and circ_0082096, were identified in the spheroid cells (Table 2). Based on sequence alignment, circ_0066631, with a predicted size of 366 nucleotides, is derived from exons 2–3 of the host transcript, DCBLD2 (CUB and LCCL domain containing 2; NM_080927), which harbors sixteen exons; likewise, the 1873-nucleotide circ_0082096 is derived from exons 4 and 5 of the host transcript zinc-finger protein 800 ((ZFN800); NM_176814) (data not shown). Very little is known about the two circRNAs, except for a few reports briefly mentioning possible involvements in viral infection and atrial fibrillation [57,58]. For the host transcripts, the highly conserved DCBLD2 protein acts as a crucial co-receptor in signaling in the processes of tumorigenesis and development (reviewed by [59]). Clinically, DCBLD2 up-regulation has been associated with tumor progression and poor prognosis in CRC patients [60,61,62]. The reported up-regulated transcription of DCBLD2 in CRC is in agreement with up-regulated levels of the derived circ_0066631 in the cancer stem cells of CRC reported in this work. On the other hand, the gene product of the host transcript ZNF800 has only been reported as a candidate master regulator in adipose gene expression and cardio-metabolic traits [63]; ZNF800 involvement in cancers has yet to be demonstrated. The addition of circ_0066631 and circ_0082096 to the short list of circRNAs involved in regulating cancer stemness accentuates the relevance of circRNAs in relation to CSC features (reviewed by [64]). 

Using integrative algorithms, circ_0066631 and circ_0082096 were predicted to target five miRNAs in stemness regulation. The experimental demonstration of the down-regulation of the five core miRNAs in the CRC spheroids (Figure 5B) agreed well with up-regulated expression and the predicted sponging functions of the two circRNAs in the spheroid CSC cells. Furthermore, the observed reversion of the core miRNA expression levels on differentiation, and, therefore, the loss of stemness properties (Figure 2B), further support that the five core miRNAs identified are regulators of CSC stemness properties. Except for miR-224, the other four miRNAs have previously been reported to be tumor suppressors [65,66,67,68]. Hence, circRNA knockdown of these tumor suppressor miRNAs shown here (Figure 5B) is consistent with involvement of circ_0066631 and circ_0082096 in the tumorigenesis process. By suppressing the translation of various targeted transcripts, the five core miRNAs have been reported to be associated in inhibiting various CSC properties, including EMT and cancer cell migration and invasion, proliferation and apoptosis, and chemoresistance (see Suppl. Table S4 for a full list of literature citations). The circRNA sponging of the core miRNAs, which are themselves negative regulators, would have enhanced CSC features in the CRC spheroidal culture via up-regulated expression of the miRNA-targeted transcripts. 

**Figure 6:**
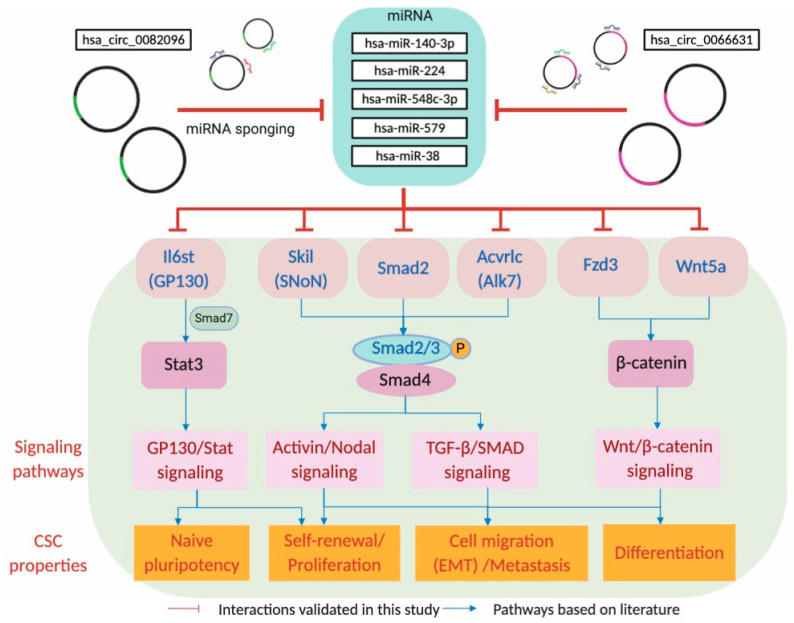
Circ_0082096 and circ_0066631 target signaling pathways that modulate known CSC properties. In the top part of the scheme, the two circRNAs are shown to sponge the five miRNAs, which in turn, modulate the respective signaling pathways (shown in pink boxes) via the gene products of the predicted miRNA-targeted transcripts (shown in red letters), subsequently resulting in the modulation of known CSC properties (orange boxes). See Discussion section for further description of the scheme.

The KEGG analysis identified six core mRNAs, ACVR1C/ALK7, FZD3, IL6ST/GP130, SKIL/SNON, SMAD2 and WNT5A, that were collectively targeted by the circRNA–miRNA axis in the regulation of pluripotency. The core mRNA levels were up-regulated in the spheroid cells and were suppressed upon serum-induced differentiation (Figure 5C and Supplementary Figure S1B), linking expression with a key stemness property. The six core mRNAs have, indeed, also previously been reported to be involved in diverse CSC-related properties, ranging from proliferation, migration and invasion, chemoresistance and tumorigenicity (see Table 4 for full references). Importantly, pluripotency-associated properties have also been demonstrated in other reports [69,70,71,72,73]. The six core mRNAs may further be implicated to be linked with four major signaling pathways that modulate numerous CSC-associated properties observed in the CRC spheroids (Figure 6). IL6ST/GP130 is an integral part of the GP130/Stat signaling pathway, interacting with Stat3 to induce naïve pluripotency and self-renewal [69,74,75]. SKIL/SNON and ACVRLC/AKT7, acting via the Activin/Nodal and TGF-β signaling pathways, are involved in the phosphorylation and association of SMAD2/3 with SMAD4 to enter the nucleus to exert transcriptional activation functions on target genes [70,76,77]. FZD3 and WNT5a regulate β-catenin in the Wnt/β-catenin signaling to contribute to self-renewal, EMT-associated biological processes and differentiation [78,79,80]. 

Detection of miRNAs and proteins in circulating cells of cancer patients in so-called blood biopsies has been widely reported and used [81]. More recent studies have also shown the detection of circRNAs in the plasma, correlating to various colorectal cancer stagings [82,83]. Since circRNA is an upstream regulator of miRNA and mRNA, it may be possible to develop a circRNA–miRNA–mRNA panel, possibly including the hsa_circ_0006631 and hsa_circ_0082096 described in this work, for clinical applications as a precision biomarker in the diagnosis and prognosis of colorectal cancer progression.

## 4. Materials and Methods

### 4.1. Cell Lines and Cell Culture

Colorectal cancer (CRC) cell lines HCT-15 and WiDr were purchased from American Type Culture Collection (ATCC). HCT-15 was cultured in DMEM high glucose (Gibco, Gaithersburg, MD, USA), while WiDr was cultured in MEM (Gibco). All CRC cells were authenticated using STR profiling. A normal human colon epithelial cell line, CRL-1790, also purchased from ATCC, was cultured in Dulbecco’s modified Eagle’s Medium (DMEM) F-12 (Gibco). Human bone marrow-derived mesenchymal stem cells (BM-MSC), obtained from Cryocord Sdn Bhd (Malaysia), was cultured in DMEM F-12. All media were supplemented with 10% fetal bovine serum (FBS) (Gibco) and 1% penicillin–streptomycin (Gibco). The cells were cultured under standard culture conditions at 37 °C in 5% CO_2_.

### 4.2. Spheroid Cell Culture

CRC cell lines were trypsinized in 0.25% trypsin-EDTA (Gibco) and suspended at a density of 5 × 10^4^ cells/mL in flasks pre-coated with 0.24% polyhydroxyethylmethacrylate (polyHEMA) (Sigma-Aldrich, St. Louis, MO, USA) in DMEM-F12 supplemented with 0.24% (*v*/*v*) methylcellulose (Sigma-Aldrich), 1× B27 supplement (Gibco), 20 ng/mL of epidermal growth factor (EGF, Miltenyi Biotec, Germany), 10 ng/mL of basic fibroblast growth factor (bFGF) (Miltenyi Biotec), 0.4% (*v*/*v*) of bovine serum albumin (BSA) (Nacalai Tesque, Japan) and 1 mg/mL of insulin (Gibco). The medium was topped up on day 7. The spheroids formed were collected by centrifugation on day 14, dissociated using Accutase (Gibco) and seeded in new flasks under the same culture conditions for propagation. 

### 4.3. Immunofluorescence Analysis

Evaluation of expression of the colorectal cancer stem cell (CrCSC)-associated markers, CD133, CD44 and ALDH1 (Cell Signaling Technology, USA), was done using immunofluorescence analysis. The passage 5 (P5)-derived spheroid cells were fixed with 4% paraformaldehyde and embedded in parafilm wax for sectioning. After blocking with 5% bovine serum albumin, the cells were stained overnight with anti-CD133, -CD44 or -ALDH1 antibodies conjugated with Alexa Fluor 488 (Invitrogen, USA). The nuclei were counterstained with DAPI. The cells were photographed under an inverted fluorescence microscope. For quantitative analysis, ImageJ was used to calculate the fluorescent intensity; the relative ratio to DAPI staining was determined [98]. 

### 4.4. Colony Forming

To assess the clonogenic ability of the CrCSC cells, the colony forming assay was performed using a serum-free methylcellulose-based medium, MethoCult™ (Stemcell Technologies, Canada) based on manufacturer’s instructions. Briefly, 1 × 10^5^ cells were seeded in MethoCult in 35-mm dishes supplemented with 20 ng/mL EGF, 10 ng/mL bFGF, 1× B27, 0.4% BSA, and 1 mg/mL insulin. The dishes were incubated at 37 °C in 5% CO_2_ in a humidified incubator for 10 days. On day 10, images of the colonies formed were captured using an inverted phase contrast light microscope (Nikon, Japan).

### 4.5. Migration and Invasion Assay

A total of 2 × 10^5^ cells/mL of parental or spheroid cells were resuspended in serum-free DMEM F-12 medium and seeded on the top chamber of a FluoroBlok transwell insert. Serum-containing DMEM F-12 medium was added on the lower chamber as a chemoattractant. For invasion assays, the insert was precoated with 7 mg/mL Geltrex LDEV-free reduced growth factor basement membrane matrix (Thermo Fisher Scientific, Waltham, MA, USA). After overnight incubation, the medium was aspirated from the top chamber and washed twice with phosphate-buffered saline. The cells were fixed with ice-cold methanol for 1 h at room temperature. The cells were stained with propidium iodide for an hour before visualizing under an inverted fluorescence microscope. Migrated and invaded cells were counted using ImageJ software.

### 4.6. Spontaneous and Lineage-Directed Differentiation Assay

Spontaneous differentiation induced by serum was performed on the passage 5 (P5)-derived spheroid cells dissociated as described above and seeded in DMEM F-12 supplemented with 10% FBS and 1% penicillin–streptomycin on treated culture flasks, as for the culture of the parental CRC cells. To assess lineage-directed differentiation ability, the CRC parental and P5 spheroid cells, seeded on Matrigel-coated dishes, were grown to 70% confluency before induction to differentiate to ectodermic (adipocyte) and mesodermic (osteocyte) lineages. The ectoderm induction medium consisted of DMEM-F12 supplemented with 10% knockout serum replacement (Gibco), 1% penicillin–streptomycin, 500 ng/mL Noggin (R&D Systems, USA) and 250 ng/mL bone morphogenetic protein 4 (BMP4) (R&D Systems). The adipogenic induction medium consisted of DMEM-F12, 4% FBS, 1% penicillin–streptomycin, 1 μM dexamethasone (Santa Cruz Biotechnology Inc, Dallas, TX, USA), 0.5 mM 3-isobutyl-1-methyl-xanthine (Sigma-Aldrich), 0.2 mM indomethacin (Sigma) and 0.1 mg/mL insulin (Gibco). Osteogenic-induction medium consisted of DMEM-F12, 4% FBS, 1% penicillin–streptomycin, 100 nM dexamethasone, 50 μg/mL ascorbate-2-phosphate (Sigma) and 10 mM β-glycerophosphate (Sigma). The medium was changed every three days. The cells were grown for seven days under the ectoderm-induction medium, and thirty days under osteogenic- and adipogenic-induction media. The lipid droplets and calcium mineralization were visualized using Oil Red O and Alizarin Red staining, respectively. 

### 4.7. Chemosensitivity Assay

The chemoresistance of CrCSC cells in the spheroids was evaluated using standard drug sensitivity protocols [99]. Briefly, the CRC parental and spheroid cells were seeded at 5 × 10^3^ cells/mL in flat-bottom 96-well culture plates (BD Biosciences, San Jose, CA, USA) and polyHEMA-coated flat-bottom 96-well culture plates (BD Biosciences), respectively, in their respective medium. The cells were incubated for 24 h at 37 °C in 5% CO_2_ in a humidified incubator. The cells were treated with drug concentrations ranging from 100 to 1000 μg/mL, or treated with sterile deionized water as a negative control. The cells were incubated further for 48 h before drug sensitivity analysis using a cell proliferation kit (CCK-8, Dojindo, Japan) according to the manufacturer’s instructions. After adding the CCK-8 solution, the plates were incubated for 2.5 h at 37 °C in the dark. The absorbance of the wells was read at 450 nm. The IC_50_ was determined using the GraphPad Prism software (GraphPad Software Inc., San Diego, CA, USA). Three independent experiments were performed.

### 4.8. Stem-Loop RT-qPCR

Predicted miRNAs were validated using stem-loop qRT-PCR [100]. Stem-loop, forward and universal reverse primers are listed in Supplementary Table S1. Following RNA extraction, 1 μg RNA was reverse transcribed with SuperScript III Reverse Transcriptase (Invitrogen, Carlsbad, CA, USA) with stem-loop primers. The resulting cDNA was subjected to quantitative real-time RT-PCR using SYBR^®^ Select Master Mix (Thermo Fisher Scientific) in QuantStudio™ 3 Real-Time PCR System (Applied Biosystems, Foster City, CA, USA). U6 was used a housekeeping control.

### 4.9. Western Blot Analysis

Cells were lysed in RIPA buffer supplemented with a protease inhibitor (Merck Millipore, Billerica, MA, USA). The protein lysates were quantified and subjected to SDS-PAGE, followed by electroblotting onto PVDF membrane. The membranes were incubated with anti-KLF4, -NANOG, -c-MYC or glyceraldehyde 3-phosphate dehydrogenase (GAPDH) antibodies (Cell Signaling Technology, Danvers, MA, USA). The reactive bands were visualized by chemiluminescence detection reagents (Merck Millipore).

### 4.10. RNA Extraction and circRNA Profiling

Total RNA was extracted using TRIzol reagent (Thermo Fisher Scientific). The RNA quality was assessed by Agilent Bioanalyzer 2100 (Santa Clara, CA, USA); samples with RNA integrity numbers higher than 9 were used for circRNA profiling. rRNA was depleted according to the manufacturer’s instructions, and subjected to RNase R (Epicenter, Madison, WI, USA) treatment to remove linear RNA and enrich circRNA following manufacturer’s instructions. The RNA was fragmented using divalent cations under elevated temperatures. The library preparation was carried out using TruSeq Stranded Total RNA Library Prep Gold (Illumina, San Diego, CA, USA). Sequencing of the library was performed on the Illumina Hiseq^TM^ 2500 system based on the manufacturer’s protocol.

### 4.11. Bioinformatic Analysis

CircRNA junction reads were identified using the find_circ software [33]. Differential expression analysis was assessed using the DEGseq algorithm. The p-value of differentially expressed circRNAs were adjusted using the Benjamini–Hochberg approach. The miRNA prediction based on the binding site in circRNAs was performed using CircInteractome [101]. CircRNA–miRNA binding prediction with a context score percentile higher than 90 was selected. The prediction of mRNA targets of selected miRNAs was performed using the miRWalk database. Cytoscape software [102] was used to illustrate the circRNA–miRNA–mRNA regulatory interaction network. Functional annotation of host genes of selected circRNAs and the predicted mRNA were analyzed using gene ontology (GO) and KEGG pathway using DAVID (version 6.8).

### 4.12. Selection of Top Four Up- and Down-Regulated circRNAs

Based on the NGS sequencing data of the parental and spheroid cells of HCT-15 and WiDr, ~15,000 circRNAs were first collectively identified from the two cell lines. Using an Orange Data Visualizing algorithm (https://orange.biolab.si/), the top four up- and down-regulated circRNAs that were differentially expressed in the parental and spheroid cells of both HCT-15 and WiDr cells were next identified from the 8262 differentially expressed circRNA dataset. For the top four up-regulated circRNAs, the list of the circRNAs was narrowed down by uniformly increasing the fold-change difference between the parental and spheroid cells of HCT-15 and WiDr. Starting from a log_2_ fold-change value of 1, we gradually increased the search values until the list was shortened to contain only four candidate circRNAs that were consistently up-regulated in the HCT-15 and WiDr spheroid cells. Likewise, the identification of the top four down-regulated circRNAs was performed by uniformly decreasing the log_2_ fold-change until only four candidate circRNAs were obtained. Using this algorithmic approach, the circRNAs identified showed a consistent trend of either up- or down-regulated circRNA expression in the HCT-15 and WiDr spheroids relative to the parental cells.

### 4.13. Quantitative Real-Time RT-PCR

Total RNA, isolated using Trizol reagent (Qiagen, Hilden, Germany), were reverse transcribed to cDNA using the SuperScript III Reverse Transcriptase (Invitrogen) using random hexamer primers, according to the manufacturer’s instruction. The cDNA was subjected to quantitative real-time RT-PCR using SYBR^®^ Select Master Mix (Thermo Fisher Scientific) in the QuantStudio™ 3 Real-Time PCR System (Applied Biosystems). Sequences of the primers used are listed in Supplementary Table S1. The expression levels were normalized to those of the glyceraldehyde 3-phosphate dehydrogenase (GAPDH) gene. Relative RNA expression levels were calculated using the comparative C_T_ (ΔΔC_T_) method. Three independent experiments were performed.

## 5. Conclusions

A circRNA–miRNA–mRNA-signaling regulatory axis has been mapped in CSC-enriched CRC spheroid cells in this work (Figure 6). In the network thus mapped, hsa_circ_0066631 and hsa_circ_0082096 act as the drivers to first knockdown five tumor suppressor miRNAs via sponging, thus releasing the six mRNA targets that are involved primarily in signaling pathways in the establishment and maintenance of stemness in reprogrammed CRC cancer stem cells. The proposed stemness-regulating circRNA–miRNA–mRNA-signaling axis in the CSC of colorectal cancer cells forms the foundation for further investigations on the molecular mechanisms and functional roles of hsa_circ_0066631 and hsa_circ_0082096 in the propagation of cancer stemness. Hsa_circ_0066631 and hsa_circ_0082096 may serve as therapeutic targets in designing differentiation therapy to eradicate cancer stem cells in colorectal cancer [103]. 

## Figures and Tables

**Table 1:** Top four differential expressed circRNAs in the spheroidal CRC.

CircRNA ID	HCT-15		Fold Change (log_2_)	*p*-Value	WiDr		Fold Change (log_2_)	*p*-Value
	Parental	Spheroid			Parental	Spheroid		
**Upregulated circRNA**								
hsa_circ_0002970	0	70.041	6.96	7.34 × 10^−6^	31.892	84.479	1.39	0.0440
hsa_circ_0008599	43.960	139.655	1.63	0.0017	15.380	67.332	2.10	0.0030
hsa_circ_0066631	37.906	117.341	1.62	0.000066	21.074	83.910	2.00	0.0011
hsa_circ_0082096	5.878	38.815	2.60	0.0030	9.682	47.797	2.26	0.0130
**Downregulated circRNA**								
hsa_circ_0000400	445.113	121.834	−1.83	0.0085	488.659	149.084	−1.70	2.21 × 10^−8^
hsa_circ_0005174	19.487	8.0366	−1.20	0.6087	11.391	1.455	−2.54	0.6759
hsa_circ_0005507	107.582	29.003	−1.87	7.57 × 10^−6^	17.089	3.568	−2.08	0.5388
hsa_circ_0040238	138.420	65.182	−1.08	0.0051	37.588	11.649	−1.59	0.7378

**Table 2:** CircRNA–miRNA–mRNA regulatory axis identified.

CircRNA	miRNA	mRNA					
		ACVR1C	FZD3	IL6ST	SKIL	SMAD2	WNT5A
hsa_circ_0066631	miR-140-3p	−	−	−	+	−	+
	miR-224	−	+	−	−	+	+
	miR-548c-3p	+	+	−	+	+	+
	has-miR-579	−	+	+	+	−	−
hsa_circ_0082096	miR-140-3p	−	−	−	+	−	+
	miR-382	+	+	+	−	−	+
	miR-579	−	+	+	+	−	−

“+”, predicted binding; “−”, no predicted binding.

**Table 3:** Predicted circRNA–miRNA binding activity.

CircRNA miRNA	CircRNA—miRNA Pairing	Minimal Free Energy (kcal/mol)	Binding Pairing
hsa_circ_0082096 miR-579	5′-AGGGAACAAAUCAUUCAAAUGAA-3 ′3′-UUAGCGCCAAAUAUGGUUUACUU-5′	−18.9	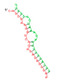
hsa_circ_0082096 miR-382	5′-AACUAAACAACUUA-3′ 3′-GCUUAGGUGGUGCUUGUUGAAG-5′	−22.6	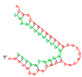
hsa_circ_0082096 miR-140-3p	5′-UGAUACGACAUAUAACUGUGGUU-3′ 3′-GGCACCAAGAUGGGACACCAU-5′	−21.6	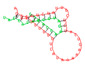
hsa_circ_0066631 miR-140-3p	5′-UGAAAUAGGCAAAUACUGUGGUC-3′ 3′-GGCACCAAGAUGGGACACCAU-5′	−18.8	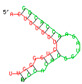
hsa_circ_0066631 miR-224	5′-UUCGCAUCAAAUUUGGUGACUUU-3′ 3′-UUGCCUUGGUGAUCACUGAAC-5′	−19.0	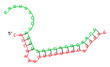
hsa_circ_0066631 miR-548c-3p	5′-GGCAAAUACUGUGGUCUGGGGUUG-3′ 3′-CGUUUUCAUUAACUCUAAA-5′	−18.0	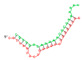
hsa_circ_0066631 miR-579	5′-UGUGGUCUGGGGUUGCAAAUGAA-3′ 3′-UUAGCGCCAAAUAUGGUUUACUU-5′	−24.1	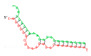

Predicted complementary binding site of circRNA (red) against the putative target miRNA (green).

**Table 4:** Involvement of the core mRNA in CSC-related properties and the implicated signaling pathways.

mRNA (Alias)	Signaling Pathway	CSC-Related Properties	References
ACVR1C (ALK7)	TGF-β/Activin/Nodal/Smad2	Proliferation and invasion	[77,84]
FZD3v (Fz-3)	Wnt/β-catenin	Proliferation	[79]
IL6ST (Gp-130)	Stat3 signaling	Tumorigenesis initiation	[85]
	Stat3/Wnt/β-catenin	Tumorigenesis initiation	[86]
	Jak/Stat3 and PI3K/AKT/mTOR	Chemoresistance	[87]
	Stat3	Naïve pluripotency	[69]
	Jak/Stat3	Self-renewal	[75]
SKIL (SnoN)	TGF-β	Chemoresistance	[88]
	TGF-β	Tumorigenesis	[89]
	PI3K/AKT	Cancer cell proliferation	[90]
	Activin/Nodal	Pluripotency maintenance	[70]
	TGF-β	Epithelial-mesenchymal transition	[91]
SMAD2	TGF-β	Pluripotency maintenance	[71,72]
	TGF-β/Akt	Stemness maintenance	[92,93]
	TGF-β/Smad/Snail	Epithelial-mesenchymal transition	[94]
	Nodal/Activin	Pluripotency maintenance	[73]
Wnt5A	TGF-β	Migration and invasion	[95]
	TGF-β	Migration and self-renewal	[96]
	TGF-β/Smad	Tumorigenesis and migration	[97]

## Supplementary Materials

Supplementary Materials can be found at https://www.mdpi.com/1422-0067/21/21/7864/s1. Figure S1. Expression reversal of core miRNAs and mRNAs upon serum-induced differentiation of the spheroid cells. Expression levels of the identified core miRNAs (A) and mRNAs (B) in the spheroid-differentiated cells were determined using qRT-PCR. * *p* < 0.05 and ** *p* < 0.01 were relative to the parental CRC cells. Table S1. List of primers used in the study. Table S2. Interactions of the top eight circRNAs with the core miRNAs (see also Figure 4E). Table S3. List of mRNAs targeted by the core miRNAs as displayed in Figure 4F. Table S4: Targeted miRNAs and affected functions: summary of literature review.

## Abbreviations

3D	three dimensional
5-FU	5-fluorouracil
ALDH1	aldehyde dehydrogenase 1
ATCC	American Type Culture Collection
bFGF	basic fibroblast growth factor
BM-MSC	bone marrow-derived mesenchymal stem cells
BMP4	bone morphogenetic protein 4
BSA	bovine serum albumin
circRNA	circular RNA
CRC	colorectal cancer
CrCSC	colorectal cancer stem cells
CSC	cancer stem cells
DMEM	Dulbecco’s modified Eagle’s Medium
EGF	epidermal growth factor
FBS	fetal bovine serum
GAPDH	glyceraldehyde 3-phosphate dehydrogenase
GO	gene ontology
KEGG	Kyoto Encyclopedia of Genes and Genomes
L-OHP	oxaliplatin
lincRNA	long intervening non-coding RNA
miRNA	microRNA
polyHEMA	polyhydroxyethylmethacrylate.
