# Supplementary material for: Mapping a Circular RNA–microRNA–mRNA-Signaling Regulatory Axis that Modulates Stemness Properties of Cancer Stem Cell Populations in Colorectal Cancer Spheroid Cells
Source: Int J Mol Sci. 2020 Oct 23;21(21):7864. doi: 10.3390/ijms21217864 (PMC7672619; doi:10.3390/ijms21217864)
Supplement: Supplementary file 1 [file ijms-21-07864-s001.zip › ijms-942229-supplementary/Suppl. Table S3 (Final).docx]

**Supplementary Table S3.** List of mRNAs targeted by the core miRNAs as displayed in Figure 4F

| AAK1 | ANGEL2 | CNTLN | ELK4 | GK5 | KCNN3 | MMP16 | PIAS2 | RNF125 | SLC7A11 | UBE2G1 |
| --- | --- | --- | --- | --- | --- | --- | --- | --- | --- | --- |
| ABHD5 | ARCN1 | CNTN3 | ENTPD4 | GNB4 | KLF12 | NAP1L1 | PLEKHA8 | RORA | SMAD2 | VAMP4 |
| ABI2 | ATXN1 | CREBRF | ETNK1 | GTF2H5 | KLHL15 | NFAT5 | PRKAA2 | RP2 | SNTB2 | WNT5A |
| ACOX1 | ATXN3 | CSRNP3 | EXOC5 | HAUS3 | KPNA1 | NOX4 | PRLR | SATB2 | ST8SIA3 | ZBTB34 |
| ACVR1C | BRWD1 | CYP20A1 | FAM129A | HIPK2 | LCOR | NPY2R | PRTG | SCAI | SYT4 | ZC3H12C |
| ADAM22 | C11orf58 | DCP2 | FAM46A | HOOK3 | MAPK9 | NTRK2 | PTAR1 | SESTD1 | TET3 | ZC3H6 |
| ADAMTS5 | C12orf5 | DCUN1D5 | FAXC | HS6ST3 | MBNL3 | NUFIP2 | PURB | SH3TC2 | TMEM56 | ZFP14 |
| ADRBK2 | C5orf24 | DOK6 | FBXW2 | IL6ST | MBTPS2 | OCLN | QKI | SKIL | TMOD2 | ZFP91 |
| AFF4 | CAMK2D | DSEL | FUT9 | IMPAD1 | MCTS1 | ONECUT2 | QSER1 | SLC1A2 | TMTC3 | ZNF587 |
| ALG10B | CHM | EEA1 | FZD3 | INO80D | MEF2A | PAK2 | RAB22A | SLC1A3 | TNRC6B | ZNF618 |
| AMMECR1 | CNOT6L | EIF4E | GABRG1 | ITPRIPL2 | MGAT4A | PDK3 | RND3 | SLC25A36 | UBA6 | ZNF704 |

mRNAs that are modulated by hsa_circ_0066631 and hsa_circ_0082096 via miRNAs are highlighted in gray.
