# Supplementary material for: Mapping a Circular RNA–microRNA–mRNA-Signaling Regulatory Axis that Modulates Stemness Properties of Cancer Stem Cell Populations in Colorectal Cancer Spheroid Cells
Source: Int J Mol Sci. 2020 Oct 23;21(21):7864. doi: 10.3390/ijms21217864 (PMC7672619; doi:10.3390/ijms21217864)
Supplement: Supplementary file 1 [file ijms-21-07864-s001.zip › ijms-942229-supplementary/Suppl. Table S4 (Final).docx]

**Supplementary Table S4.** Targeted miRNAs and affected functions: summary of literature review

| **miRNA** | **Identified target(s)** | **Affected functions** | **Cancer** | **References** |  |
| --- | --- | --- | --- | --- | --- |
| miR-548c-3p | None  NRIP1  ECHS1 | Tumor suppressor  Enhances the migration and invasion  Inhibits proliferation and promote apoptosis | Lung  Esophageal  Breast | [1]  [2]  [3] | |
| miR-579-3p | mTOR, Rheb & PDK1  MSR1  BRAF & MDM2 | Tumor suppressor  STAT3-mediated chemoresistance  Controls cancer progression and drug resistance | Glioblastoma  Melanoma  Melanoma | [4]  [5]  [6] | |
| miR-382 | NR2F2  BDNF  SP1  KLF12 & HIPK3 | Inhibits cell growth and invasion  Inhibits cell proliferation and invasion  Inhibits cell growth and migration  Inhibited cell proliferation, migration, invasion & enhance chemosensitivity | Colorectal  Retinoblastoma  Colorectal  Colorectal | [7]  [8]  [9]  [10] | |
| miR-140-3p | BRD9  ATP8A1  ADAMTS5 & IGFBP5  SMAD3  PD-L1 | Tumor suppressor  Inhibits cell growth and invasion  Inhibits cancer stem cell survival and invasive potential  Inhibits colorectal cancer invasion and metastasis  Suppresses cell growth; induces apoptosis | Lung  Lung  Colorectal  Colorectal  Colorectal | [11]  [12]  [13]  [14]  [15] | |
| miR-224 | USP3 & SMAD4  GSK3β & SFRP2  KRAS  SMAD4 | Associated with metastasis  Tumor progression  Cancer progression and chemoresistance  Promotes cell proliferation | Colorectal  Colorectal  Colorectal  Colorectal | 16]  [17]  [18]  [19] | |

**References:**

1. Wang, Z.; Wu, X.; Hou, X.; Zhao, W.; Yang, C.; Wan, W.; Chen, L. miR-548b-3p functions as a tumor suppressor in lung cancer. *Lasers Med. Sci.* **2019**.

2. Ni, X.F.; Zhao, L.H.; Li, G.; Hou, M.; Su, M.; Zou, C.L.; Deng, X. MicroRNA-548-3p and microRNA-576-5p enhance the migration and invasion of esophageal squamous cell carcinoma cells via NRIP1 down-regulation. *Neoplasma* **2018**, *65*, 881–887.

3. Shi, Y.; Qiu, M.; Wu, Y.; Hai, L. MiR-548-3p functions as an anti-oncogenic regulator in breast cancer. *Biomed. Pharmacother.* **2015**, *75*, 111–116.

4. Kalhori, M.R.; Irani, S.; Soleimani, M.; Arefian, E.; Kouhkan, F. The effect of miR-579 on the PI3K/AKT pathway in human glioblastoma PTEN mutant cell lines. *J. Cell. Biochem.* **2019**, *120*, 16760–16774.

5. Wang, X.; Qu, H.; Dong, Y.; Wang, G.; Zhen, Y.; Zhang, L. Targeting signal-transducer-and-activator-of-transcription 3 sensitizes human cutaneous melanoma cells to BRAF inhibitor. *Cancer Biomarkers* **2018**, *23*, 67–77.

6. Fattore, L.; Mancini, R.; Acunzo, M.; Romano, G.; Laganà, A.; Pisanu, M.E.; Malpicci, D.; Madonna, G.; Mallardo, D.; Caponea, M.; et al. miR-579-3p controls melanoma progression and resistance to target therapy. *Proc. Natl. Acad. Sci. U. S. A.* **2016**, *113*, E5005–E5013.

7. Zhou, B.; Song, J.; Han, T.; Huang, M.; Jiang, H.; Qiao, H.; Shi, J.; Wang, Y. MiR-382 inhibits cell growth and invasion by targeting NR2F2 in colorectal cancer. *Mol. Carcinog.* **2016**, *55*, 2260–2267.

8. Song, D.; Diao, J.; Yang, Y.; Chen, Y. MicroRNA-382 inhibits cell proliferation and invasion of retinoblastoma by targeting BDNF-mediated PI3K/AKT signalling pathway. *Mol. Med. Rep.* **2017**, *16*, 6428–6436.

9. Ren, Y.; Zhang, H.; Jiang, P. MicroRNA-382 inhibits cell growth and migration in colorectal cancer by targeting SP1. *Biol. Res.* **2018**, *51*.

10. Yao, H.; Xia, D.; Li, Z. lin; Ren, L.; Wang, M. ming; Chen, W. sheng; Hu, Z. chuan; Yi, G. ping; Xu, L. MIR-382 functions as tumor suppressor and chemosensitizer in colorectal cancer. *Biosci. Rep.* **2019**, *39*.

11. Huang, H.; Wang, Y.; Li, Q.; Fei, X.; Ma, H.; Hu, R. miR-140-3p functions as a tumor suppressor in squamous cell lung cancer by regulating BRD9. *Cancer Lett.* **2019**, *446*, 81–89.

12. Dong, W.; Yao, C.; Teng, X.; Chai, J.; Yang, X.; Li, B. MiR-140-3p suppressed cell growth and invasion by downregulating the expression of ATP8A1 in non-small cell lung cancer. *Tumor Biol.* **2016**, *37*, 2973–2985.

13. Yu, L.; Lu, Y.; Han, X.; Zhao, W.; Li, J.; Mao, J.; Wang, B.; Shen, J.; Fan, S.; Wang, L.; et al. microRNA -140-5p inhibits colorectal cancer invasion and metastasis by targeting ADAMTS5 and IGFBP5. *Stem Cell Res. Ther.* **2016**, *7*, 1–11.

14. Li, J.; Zou, K.; Yu, L.; Zhao, W.; Lu, Y.; Mao, J.; Wang, B.; Wang, L.; Fan, S.; Song, B.; et al. MicroRNA-140 inhibits the epithelial-mesenchymal transition and metastasis in colorectal cancer. *Mol. Ther. - Nucleic Acids* **2018**, *10*, 426–437.

15. Jiang, W.; Li, T.; Wang, J.; Jiao, R.; Shi, X.; Huang, X.; Ji, G. miR-140-3p suppresses cell growth and induces apoptosis in colorectal cancer by targeting PD-L1. *Onco. Targets. Ther.* **2019**, *12*, 10275–10285.

16. Wang, Z.; Yang, J.; Di, J.; Cui, M.; Xing, J.; Wu, F.; Wu, W.; Yang, H.; Zhang, C.; Yao, Z.; et al. Downregulated USP3 mRNA functions as a competitive endogenous RNA of SMAD4 by sponging miR-224 and promotes metastasis in colorectal cancer. *Sci. Rep.* **2017**, *7*, 1–14.

17. Li, T.; Lai, Q.; Wang, S.; Cai, J.; Xiao, Z.; Deng, D.; He, L.; Jiao, H.; Ye, Y.; Liang, L.; et al. MicroRNA-224 sustains Wnt/β-catenin signaling and promotes aggressive phenotype of colorectal cancer. *J. Exp. Clin. Cancer Res.* **2016**, *35*.

18. Amankwatia, E.B.; Chakravarty, P.; Carey, F.A.; Weidlich, S.; Steele, R.J.C.; Munro, A.J.; Wolf, C.R.; Smith, G. MicroRNA-224 is associated with colorectal cancer progression and response to 5-fluorouracil-based chemotherapy by KRAS-dependent and-independent mechanisms. *Br. J. Cancer* **2015**, *112*, 1480–1490.

19. Wang, Y.; Ren, J.; Gao, Y.; Ma, J.Z.I.; Toh, H.C.; Chow, P.; Chung, A.Y.F.; Ooi, L.L.P.J.; Lee, C.G.L. MicroRNA-224 targets SMAD family member 4 to promote cell proliferation and negatively influence patient survival. *PLoS One* **2013**, *8*.
