# Supplementary material for: Mapping a Circular RNA–microRNA–mRNA-Signaling Regulatory Axis that Modulates Stemness Properties of Cancer Stem Cell Populations in Colorectal Cancer Spheroid Cells
Source: Int J Mol Sci. 2020 Oct 23;21(21):7864. doi: 10.3390/ijms21217864 (PMC7672619; doi:10.3390/ijms21217864)
Supplement: Supplementary file 1 [file ijms-21-07864-s001.zip › ijms-942229-supplementary/Suppl. Table S2 (Final).docx]

**Supplementary Table S2.** Interactions of the top eight circRNAs with the core miRNAs (see also Figure 4E)

| **miRNA** | **hsa_circ_** | | | | | | | |
| --- | --- | --- | --- | --- | --- | --- | --- | --- |
|  | **0000400** | **0002970** | **0005174** | **0005507** | **0008599** | **0040238** | **0066631** | **0082096** |
| miR-1272 | + | + | - | + | - | - | - | + |
| miR-1322 | + | + | - | + | - | - | - | + |
| miR-140-3p | + | - | - | + | - | - | + | + |
| miR-144 | + | - | - | + | - | - | + | + |
| miR-224 | + | - | + | + | - | - | + | - |
| miR-361-3p | + | + | - | + | - | + | - | - |
| miR-382 | + | - | - | + | - | + | - | + |
| miR-520g | + | - | - | + | - | + | + | - |
| miR-520h | + | - | - | + | - | + | + | - |
| miR-548c-3p | + | - | + | + | - | - | + | - |
| miR-548m | + | + | - | + | - | + | - | - |
| miR-579 | + | - | - | + | - | - | + | + |
| miR-595 | + | + | - | + | - | + | - | - |
| miR-616 | + | - | + | + | - | + | + | - |
| miR-659 | + | - | - | + | + | - | - | + |

“+”, predicted binding; “- “, no predicted binding.
