# Supplementary material for: Mapping a Circular RNA–microRNA–mRNA-Signaling Regulatory Axis that Modulates Stemness Properties of Cancer Stem Cell Populations in Colorectal Cancer Spheroid Cells
Source: Int J Mol Sci. 2020 Oct 23;21(21):7864. doi: 10.3390/ijms21217864 (PMC7672619; doi:10.3390/ijms21217864)
Supplement: Supplementary file 1 [file ijms-21-07864-s001.zip › ijms-942229-supplementary/Suppl. Table S1 (Final).docx]

**Supplementary Table S1**. List of primers used in the study

| **mRNA** | **Forward** | **Reverse** |
| --- | --- | --- |
| CD133 | GATTAAGTCCATGGCAACAGCG | GCTGGTCAGACTGCTGCTAAGC |
| CD44 | CTGCAGGTATGGGTTCATAG | ATATGTGTCATACTGGGAGGTG |
| ALDH1 | TGTTAGCTGATGCCGACTTG | CTTCTTAGCCCGCTCAACAC |
| PAX6 | ACATACCAAGCGTGTCATCAA | GCCCGTTCAACATCCTTAGT |
| NF-200 | TCCTACCAGGAAGCCATTCA | CCAGAGCCATCTTGACATTGAG |
| FZD3 | AAAGCTCGCTGTCGCTGG | GAAATGCTATCCTCAGACCCC |
| IL6ST | ACAGAACAGCATCCAGTGTCA | TCTGGAGGCAAGCCTGAAATTA |
| SKIL | TATGCAGGACAGTTGGCAGAA | TTGCTTCCCGTTCCTGTCTG |
| SMAD2 | TGTTTTCAGTTCCGCCTCCA | GCCTCTTGTATCGAACCTGC |
| ACVR1C | CCAACAGCATCACCAAATGCC | CAGCATCGCAGCTATGGACA |
| WNT5A | ACTATGGCTACCGCTTTGCC | GGTTGTACACCGTCCTGCG |
| GAPDH | CTCAACTACATGGTTTACATGTTC | TGGAAGATGGTGATGGGATT |
| U6 | GCTTCGGCAGCACATATACTAAAAT | CGCTTCACGAATTTGCGTGTCAT |
| **CircRNA** | **Forward** | **Reverse** |
| hsa_circ_0066631 | AACAAGGTGATGGATGTGGAC | TGCGAACTCTCTCTCCCATC |
| hsa_circ_0082096 | GGCTCTTGTCTGGAACTAAACA | TCTGAAGAGACTGCGGCATA |
| hsa_circ_0002970 | GCCGGTGATGTAGACGAAAG | CTATCTCCTCCCCGATGTGC |
| hsa_circ_0008599 | AATGACTGGTCTACGTGGGG | GGACCAGAAACCTGCAGTGT |
| hsa_circ_0000400 | TTCGCCTCCTAATCCCTAGC | CCGTGTTCCAGGCAGTAGA |
| hsa_circ_0005174 | GGACTTCCGGGGTAATGACA | TCTTGATGGGACCGTTTTATCC |
| hsa_circ_0005507 | AGAATTGAAGCTGCGGGGTA | GGCACTCCTTTCCCTACTGT |
| hsa_circ_0040238 | ACTTGGGTACATCTGGGGAC | GCAGACTTCCACGTTGTTCA |
| **miRNA** | **Stem-loop primer** | **Forward primer** |
| miR-140-3p | GTCGTATCCAGTGCAGGGTCCGAGGTATTCGCACTGGATACGACGGCACCA | AATACGCG TACCACAGGGTAG |
| miR-224 | GTCGTATCCAGTGCAGGGTCCGAGGTATTCGCACTGGATACGACCTAAACG | AAGCCGCGTCAAGTCACTAGT |
| miR-548c-3p | GTCGTATCCAGTGCAGGGTCCGAGGTATTCGCACTGGATACGACGCAAAAG | CGCGGCCCAAAAATCTCAAT |
| miR-579 | GTCGTATCCAGTGCAGGGTCCGAGGTATTCGCACTGGATACGACAATCGCG | ATGCGCGCTTCATTTGGTATAA |
| miR-382 | GTCGTATCCAGTGCAGGGTCCGAGGTATTCGCACTGGATACGACCGAATCC | AAGCCGCAGAAGTTGTTCGTG |
| miR-548m | GTCGTATCCAGTGCAGGGTCCGAGGTATTCGCACTGGATACGACCAAAAAC | CCGCCGCA CAAAGGTATTTGT |
| miR-616 | GTCGTATCCAGTGCAGGGTCCGAGGTATTCGCACTGGATACGACAAGTCAC | CTGCGCGAACTCAAAACCCTT |
| U6 RT primer | TGACACGCAAATTCGTGAAGC | |
| Universal reverse | GTCGTATCCAGTGCAGGGTCC | |
