# Supplementary figures and images for: Mapping a Circular RNA–microRNA–mRNA-Signaling Regulatory Axis that Modulates Stemness Properties of Cancer Stem Cell Populations in Colorectal Cancer Spheroid Cells
Source: Int J Mol Sci. 2020 Oct 23;21(21):7864. doi: 10.3390/ijms21217864 (PMC7672619; doi:10.3390/ijms21217864)

## Slide 1
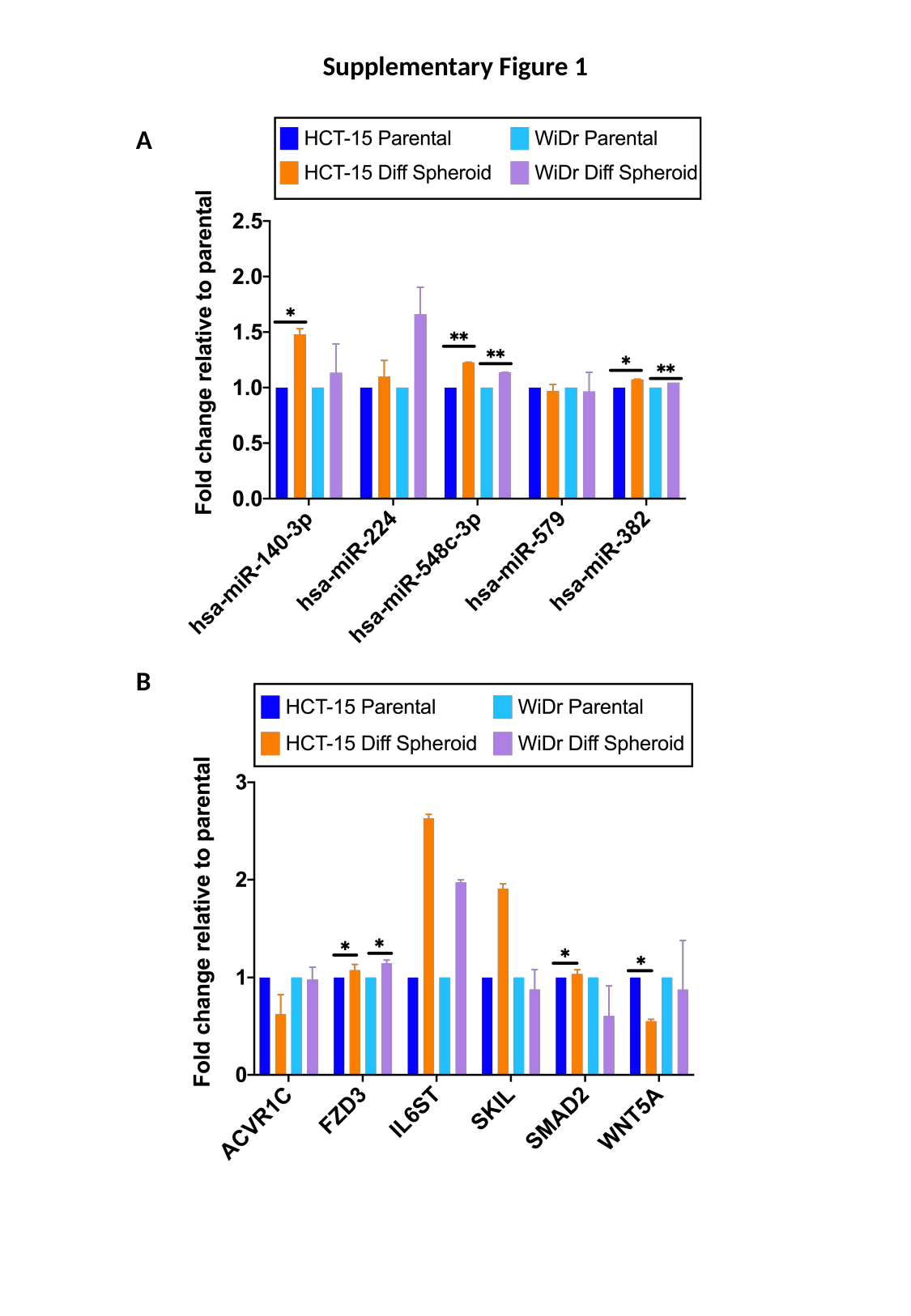

Supplementary Figure 1
A
B

Supplement: Supplementary file 1 [file ijms-21-07864-s001.zip › ijms-942229-supplementary/Supp Figure 1 (Final).pptx]
